# Methods for the health technology assessment of complex interventions: A scoping review

**DOI:** 10.1371/journal.pone.0315381

**Published:** 2025-03-14

**Authors:** Abdolvahab Baghbanian, Drew Carter, Tracy Merlin

**Affiliations:** Adelaide Health Technology Assessment, School of Public Health, University of Adelaide, Adelaide, South Australia, Australia; University of Glasgow School of Health and Wellbeing, INDIA

## Abstract

**Introduction:**

Health Technology Assessment (HTA) methods have been developed to support evidence-informed policy-making by assessing the comparative value and costs of health interventions and programs. However, the complexity of many health interventions presents challenges to the use of conventional HTA methods. This scoping review collated and synthesised international approaches to the HTA of complex interventions including identifying assessment criteria, types of evidence and the domains of value that are most favoured.

**Materials and methods:**

A systematic scoping review was conducted using JBI guidelines, Arksey and O’Malley’s six-stage framework and recent advances in scoping review methodology. Seven electronic databases, grey literature sources, three leading HTA journals and backward citation searching were used to search complex intervention HTA records written in English from January 2000 to December 2023. Supplementary searches were also conducted to identify actual HTA reports produced by certain countries. The Population (or Participants), Concept and Context framework guided the literature selection process, with a two-phase screening process and subsequent narrative synthesis. The PRISMA-ScR checklist guided reporting. Independent screening by two reviewers ensured accuracy of study selection, and data extraction followed a customised form grounded in the HTA-core model.

**Results:**

A total of 10684 references yielded 35 records from twelve countries. The review identified two clusters of research on HTA of complex interventions: methodological orientation and conceptual models (n = 19) and actual HTAs conducted on complex interventions (n = 16). Several evaluation criteria and domains were used or recommended for use that extended beyond the core HTA domains. Three distinct HTA approaches emerged: the integrative approach, highlighted in methodological guides and theoretical frameworks; and either sequential or concurrent approaches, emphasised in practical HTAs. In the theoretical literature, equal weight is given to various HTA domains for complex intervention assessment, but in practice, the scope and specificity of domains vary across reports, with countries exhibiting differing priorities. Cost-effectiveness, clinical effectiveness and organisational aspects predominated in complex intervention evaluation, albeit with gradually increasing emphasis on a technology’s description, intended use, safety and patient and social aspects over the past decade. There was less focus on ethical and legal considerations. This trend is consistent with the evaluation of non-complex interventions in HTA. HTAs undertaken on complex interventions introduced unique domains like politics, implementation, early stakeholder engagement, outcome uncertainty, adaptive methods and real-world data, with expert opinion recommended when data were insufficient.

**Conclusion:**

A shift towards considering broader contextual and implementation factors in the HTA of complex interventions was evident in this scoping review, extending beyond traditional HTA domains. However, discrepancies persist between theoretical and methodological guidance suggesting one approach and practical HTAs often adopting another. The implications of the shift towards contextual and implementation factors require exploration in future research. This could help to establish consensus on metrics and evidentiary elements, optimising HTA for complex health interventions.

## Introduction

Health Technology Assessment (HTA) methods have been developed to support evidence-based decision-making through systematic assessment of the benefits/costs and comparative value of health interventions and programs [1–3]. However, many healthcare interventions are complex in ways that pose challenges to the use of conventional HTA methods [4–6]. Examples include cell and gene therapies, screening programs and palliative care programs [see, e.g., 7].

HTA methods are well suited to pharmaceutical interventions, where causal pathways are generally understood, and specific procedures and protocols are developed for implementation. For complex interventions (CIs), however, HTA methods may face challenges and need modifying [7,8]. One critical gap in the current HTA is its limited ability to fully capture the dynamic interactions, contextual factors and long-term impacts associated with complex interventions [9]. As such, these multi-component, context-sensitive interventions require broader, adaptive methods to assess their effectiveness in real-world settings [10,11]—an area where traditional HTA may struggle.

Traditional HTA methods often rely on empirical research and clinical trials to determine an intervention’s clinical effectiveness, safety and cost-effectiveness. However, these methods may not fully consider all aspects that could influence and predict the outcome of an intervention, for example, the broader spectrum resource implications for care delivery, long-term sustainability, individual patient perspectives and preferences, the organisation and delivery of care and the cultural context. By not addressing these aspects, traditional HTA methods may provide an incomplete understanding of an intervention’s potential impact and value. This can lead to decisions that only partially reflect the healthcare system’s and its stakeholders’ needs and priorities. In particular, recognition that complex interventions are more sensitive to their context than other technologies has implications not only for the intervention’s effect but also for its acceptability, implementation and sustainability [12,13].

Complex interventions in health have no agreed definition. In the protocol underpinning this scoping review, we proposed that complex interventions characteristically include multiple components and stakeholders that interact synergistically in non-linear and dynamic pathways where histories (initial conditions) and contextual variables matter but are difficult to identify [14]. However, researchers’ understanding of complex interventions and their constituent features can vary [15–18], leading to differences in how complex interventions are defined and evaluated.

The comprehensive assessment of complex interventions can be challenging [19]. For example, comparatively little is known about how to best assess a complex intervention’s organisational, social, cultural, legal or ethical aspects [20]. These factors can play a key role in determining the outcome (success or failure) of a complex intervention when implemented or delivered in real-world conditions/settings, so it is essential to assess them properly. Besides, existing methodological literature has yet to provide thorough guidance on how to identify and assess a complex intervention’s dynamic interaction within its implementation context [21], which can significantly impact outcomes. Although HTA typically occurs before the technology is fully implemented in the health system, it is still possible to assess these contextual interactions through pilot studies, small-scale implementations, simulations, stakeholder consultations, expert opinions, modelling scenarios or review of similar interventions in comparable contexts. While difficult, these methods can provide useful information about the feasibility and potential challenges of implementing complex interventions, helping to anticipate and mitigate issues that could affect their successful integration into the healthcare system.

In addition, complex health interventions and the conditions they aim to address often present high levels of uncertainty, making it difficult to assess and utilise them effectively. This complexity can arise from different factors, including the intricate design of the intervention itself, the multi-dimensional and interconnected nature of the health conditions and the involvement of diverse stakeholder groups with differing perspectives and interests. As a result, substantial effort, collaboration and resources may be required to effectively understand, assess and implement complex interventions.

Articles critical of the HTA of complex interventions, and arguing for how complexity thinking might be helpfully applied, are starting to appear [15,22–25].

Complexity thinking can help inform the design, evaluation and implementation of complex interventions by highlighting that these interventions often consist of multiple interrelated/interconnected components and subsystems, and success may depend on their relationship with one another and with the health system and broader societal context. It provides a framework for understanding interventions as dynamic wholes rather than isolated actions or parts. This perspective emphasises the interactions and feedback loops within the environment where the intervention operates [26,27]. With complexity thinking, HTA might better understand the various drivers, outcomes and perspectives associated with complex interventions and provide a more comprehensive assessment of their implications, costs and values [8,28,29].

Complexity thinking arguably represents a paradigm shift from previous scientific approaches, including reductionism, which breaks complex systems into smaller, simpler parts, where each is studied in isolation [30,31]. The traditional view of cause and effect assumes linearity, in which the output and behaviour of a system are somehow proportional to its input. This view is based on an additive model, where the system is perceived as the sum of its components and has arguably dominated medical thought for years [30]. However, this view is limited in its ability to recognise and account for the complexity of many healthcare interventions, where the relationships between cause and effect are not easily predictable, with non-linear patterns emerging [31]. This can lead to difficulties in understanding and predicting the true impacts of complex interventions.

Nonetheless, despite the growing evidence and methodological progress in HTA, questions remain about the applicability of HTA reports on complex interventions across various jurisdictions and decision-making contexts. There is still no agreement on what constitutes a complex intervention, the evidence and evaluation criteria necessary for assessing complex interventions or how HTAs are conducted globally to evaluate complex interventions. It is, therefore, unclear what dimensions primarily determine whether an intervention should be funded.

To address these gaps, we conducted a systematic scoping review to map how complex interventions are defined in HTA (with a separate manuscript currently in preparation for publication) and determine the range of methods used to evaluate complex interventions in HTA. In particular, the scoping review collated and synthesised the published research literature to address the key question of ‘how is HTA being done by HTA agencies to formally evaluate complex interventions at the international level?’. The research sub-questions were:

1. What evaluation criteria and domains are assessed in the HTA of complex interventions?
2. Does the HTA approach differ from country to country?
3. What is the current practice in the HTA of complex interventions in Australia?

This review will help identify knowledge gaps and any variations in practice, with a view to determining where these gaps/variations should be reduced, such as through a shared definition of complex intervention or a more widely adopted approach to evaluating complex interventions. It will help HTA experts and health policy makers understand the types of information and evaluative criteria currently used in HTAs of complex interventions and thus better equip them to decide between options for conducting such HTAs.

## Materials and methods

This scoping review began with a protocol prospectively registered with the Open Science Framework (Registration DOI: https://doi.org/10.17605/OSF.IO/MU295), and its methods have been detailed elsewhere (https://doi.org/10.1136/bmjopen-2020-039263) [14].

The scoping review conforms to Arksey and O’Malley’s six-stage methodological framework [32] but features further refinements made by Levac, Colquhoun [33] and the Joanna Briggs Institute (JBI) [34]. We also drew on insights from more recent innovations in Arksey and O’Malley’s framework made by Colquhoun, Levac [35] and Tricco, Lillie [36] in setting out the plan and reporting of the review.

Seven electronic bibliographic databases of Medline/PubMed, Cochrane Library, PsycINFO, CINAHL, Embase, Epistemonikos and the INAHTA HTA database were used. In addition, a grey literature search including the manual search of three leading journals with the highest number of published papers in HTA of complex interventions; a Google search for non-indexed and unpublished literature as well as websites of HTA international, the International Society of Pharmacoeconomic Outcomes Research, and HTA agencies that are members of INAHTA were conducted. This included research in progress, theses, in-press articles, technical HTA reports, guidelines and procedure documentation, and aimed to identify guidance documents and actual reports of HTAs of complex interventions. The researchers believed all these information sources would cover all relevant literature not indexed in the above databases. The scoping review used the PCC (Population, Concept, and Context) framework to align the study selection with the research question [34]. PRISMA-ScR (The Preferred Reporting Items for Systematic Reviews and Meta-Analyses Extension for Scoping Reviews) [36,37] formed the basis for reporting findings in this scoping review.

Identifying relevant studies and information sources involved the creation of a search strategy underpinned by inclusion and exclusion criteria. These criteria were categorised under the broad PCC mnemonic recommended for scoping reviews (see Table 1).

**Table 1:** PCC framework for selection of eligible studies.

PCC Element	Definition/Determinants (per JBI Reviewer’s Manual Ch. 11)	Inclusion Criteria
**P – Population**	Documents produced by HTA agencies or HTA networks, HTA evaluators or HTA methodologists	Published studies and grey literature in the form of HTA reports or HTA guidance or methods documents. HTA reports include secondary research studies that involve systematic reviews, realist reviews, meta-analyses, meta-syntheses, mixed-methods reviews, qualitative reviews, rapid reviews with/without economic evaluations, budget impact analyses and ethical, social, legal and organisational analyses undertaken to specifically inform a health policy decision. Studies showing their full texts are available in English through academic journals, institutional repositories, archives, or other collections of scientific and other articles.
**C – Concept**	How health technology assessment* of complex interventions** is undertaken How health technology assessment of complex interventions ought to be conducted * HTA is defined at http://htaglossary.net ** complex intervention is defined at [14]	Must have a specific focus on the HTA of complex interventions in healthcare.
**C – Context**	All settings are considered. HTA must be conducted for an access or funding decision, whether at the national, regional or hospital level.	Global search for all published studies and grey literature. Search of a purposive sample of selected countries (and their respective HTA bodies) for their HTA reports and guidance documents from the INAHTA* member list (www.inahta.org) English language studies available between January 2000 and August 2020, updated again in December 2023. (Note: 2000 was chosen as the starting point for this review as it represents a period when many guidelines on HTA of complex intervention were introduced. This allowed us to capture the evolution of HTA methodology since then.

*INAHTA: The International Network of Agencies for Health Technology Assessment.

The screening and selection of eligible studies were carried out in two phases: initial screening for relevant studies based on the title and abstract, followed by a full-text review of those selected at the screening stage. The review team (AB, TM and DC) conferred to determine the reason for any differences in study selection until they reached a consensus – nearly 50 abstracts and full-texts required independent team review to decide their inclusion or exclusion.

Studies were excluded if the following conditions were met:

i. studies were published outside the specified time frame
ii. studies conducted or published in languages other than English
iii. studies where the full text could not be accessed
iv. other forms of publications, including letters, commentaries or editorials, debates, narrative reviews, study protocols, trials, as well as conference abstracts and presentations on the use of HTA of complex interventions,
v. studies without evidence of HTA of complex interventions and those non-compliant with our inclusion criteria (PCC).

A data-charting form was jointly developed by all reviewers (AB, DC and TM) to determine which variables to extract and ease the synthesising process.

The primary reviewer (AB) piloted the data extraction form with less than 10% of the included studies. The research team further examined the extracted data to verify accuracy and consistency in an iterative process. Finally, the extracted findings were summarised and mapped using narrative synthesis [38,39].

The evaluation criteria and domains were categorised using Busse’s original nine core criteria, central to HTA methodology, including to the HTA core model [40]. The HTA Core Model was initially developed as methodological guidance by the European network for HTA (known as EUnetHTA) to provide consistency and a foundation for “joint” HTA assessments across Europe [41,42]. It includes all aspects potentially relevant for HTA and thus value assessment, with the information in an ideal HTA divided into nine domains.

1. Health problems and current use of technology
2. Description and technical characteristics
3. Safety
4. Clinical effectiveness
5. Costs and economic evaluation
6. Ethical analysis
7. Organisational aspects
8. Patient and Social aspects
9. Legal aspects [40].

Based on a survey of member agencies of the International Network of Agencies for HTA, a full HTA report will always include criteria 1 to 7 [43], a mini-HTA report will always include criteria 1 to 5, and rapid reviews will always include criteria 1 to 4 from the list of nine above. Within the current scoping review, any reports/products that did not fit into one of the three preceding HTA types were labelled ‘other’ and deemed ineligible for this review.

As the primary search was aimed at finding methodological guidance documents on the HTA of complex interventions, a separate search was conducted in selected countries to identify actual reports of HTAs of complex interventions to determine what methods were applied. The search for actual HTA reports was limited to English-language full text from the last 10 years (2013–2023) to focus on recent practices and ensure relevance to current policy and decision-making contexts. We believe this distinction allowed us to explore the historical development of methodological guidance and the contemporary application of those methods.

This scoping review did not formally assess the quality of the included methodological studies [44], since the review’s main focus was not on the quality of the included studies’ findings but on the methods used.

## Results

**Fig 1:**
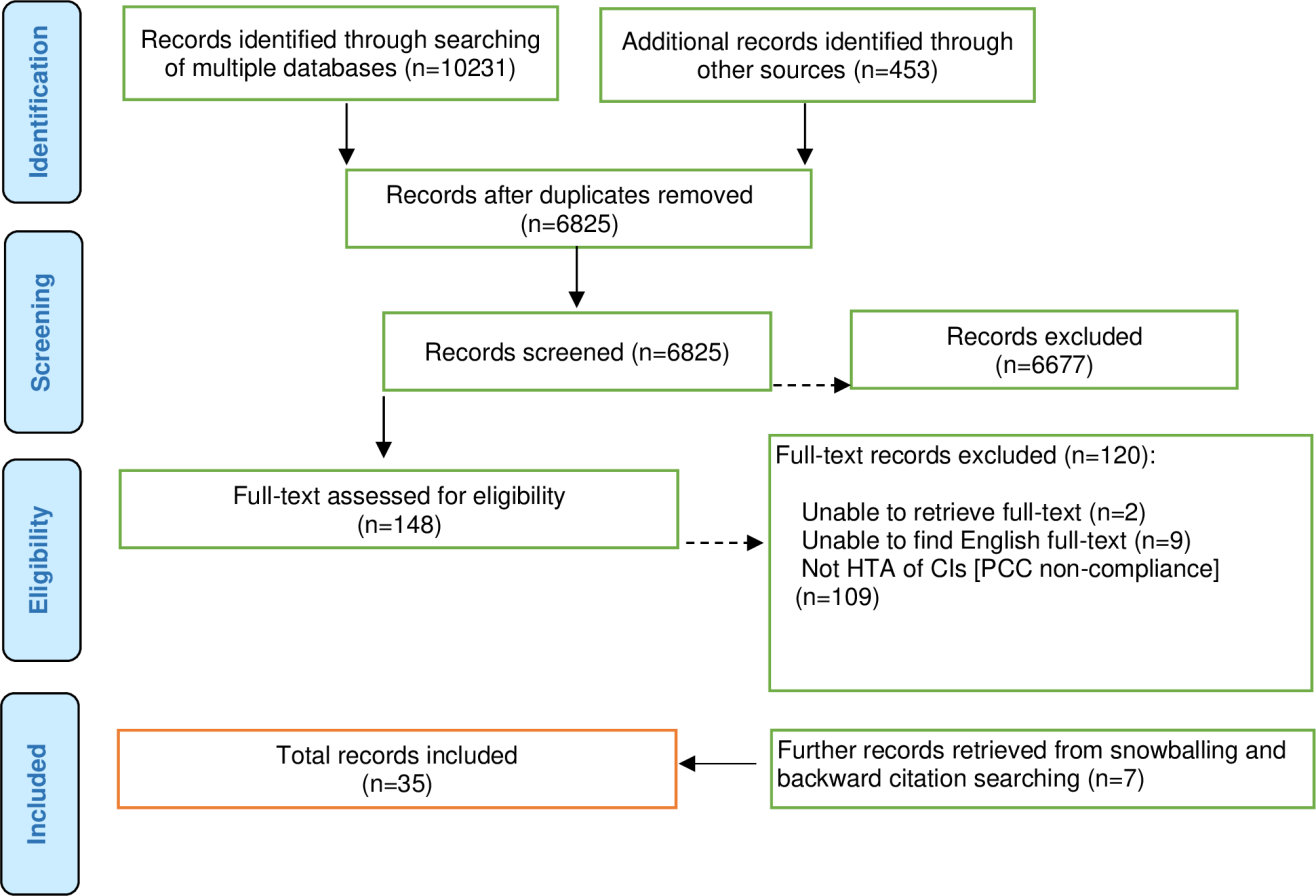
Flow diagram for the scoping review process adapted from PRISMA-ScR [36, 37].

Fig 1 shows a summary of the search and retrieval results. Thirty-five records from different countries were analysed. These were participating countries in Integrate-HTA (England, Germany, Italy, Lithuania, the Netherlands, Norway and Poland), Australia, Canada, China and some European countries, including the United Kingdom, Sweden, Belgium, Wales and Ireland. We note that empirical studies on the HTA of complex interventions were limited, and almost all of the research reported in these papers was conducted in developed countries. Furthermore, most of the selected journal articles or research papers adopt mainly an exploratory or explanatory approach rather than focusing on how the HTA of complex interventions is implemented in practice, with some focusing on conceptual or theoretical designs.

The majority of documents included in this review (n = 19) outlined how the HTA of complex interventions should be conducted (i.e., they prescribed a methodological orientation and conceptual model of the HTA of complex interventions rather than an actual representation); including records that alluded to methods and guidance used in the Integrate-HTA project (an approach to the HTA of complex interventions) [5,9,13,45–58]. In contrast, a smaller number of the included records (n = 16) described how the HTA of complex interventions is carried out in practice, including full-HTAs, an environmental scan, empirical research studies and various forms of literature reviews [7,59–70].

### Synthesis

A mind map was developed to visually set out key words and concepts identified in the publications. This was used to decide on the core domains and parameters described below.

### Evaluation criteria and domains in the HTA of complex interventions

The review identified several key domains and criteria in the HTA of complex interventions. Tables 2 and 3 show the key evaluation criteria and domains used or recommended in the HTA of complex interventions. The tables differentiate between methodological or guidance documents (19 papers) and actual or practice studies (16 studies). They provide an overview of the key assessment criteria and domains considered within each category to gain insights into the focus and emphasis of the HTA approaches utilised or prescribed in the field.

**Table 2:** Recommended evaluation criteria and domains in HTA of complex interventions (adapted from Established Guidance Documents and Theoretical Frameworks).

Source & Country	Approach	HTA Product or Dissemination Method	Evaluation Criteria, Domains or Evidence Types Based on the HTA Core Model									Other consideration
			Health problem & current use of technology	Description & Technical Characteristics of technology	Safety	Clinical Effectiveness	Cost & Economic Evaluation	Ethical	Organisational	Patient & Social	Legal	
Gerhardus [5] Integrate-HTA Countries.	T/G	Introduction to guidances										Political, environmental and geographical factors, early stakeholder input, interactions, relevant evidence
Booth, Noyes [45] Integrate-HTA Countries.	T/G	Guidance										Qualitative evidence synthesis, early stakeholder input, interactions
Pfadenhauer, Rohwer [51] Integrate-HTA Countries.	T/G	Guidance										Political, environmental and geographical factors, intervention design and implementation, early stakeholder input, interactions, relevant evidence.
Pfadenhauer, Gerhardus [71] Integrate-HTA Countries.	T/G	Journal Article										Political, environmental and geographical factors, early stakeholder input, interactions, available evidence, advocacy groups, intervention sustainability.
Wahlster, Brereton [72] Integrate-HTA Countries.	T/G	Guidance										Modifiers, uncertainty, stakeholder representation, geography, interactions, quality evidence
Wahlster, Brereton [57] Integrate-HTA Countries.	T/G	Journal Article (Theme Submission)										Political and geographical factors, intervention design and implementation, early stakeholder input, interactions, relevant evidence, qualitative and quantitative methods, Logic models, HTA model flexibility.
Rohwer, Booth [9] Integrate-HTA Countries.	T/G	Guidance										Geographical factors, intervention delivery, early stakeholder input, interactions, relevant evidence, Logic models, global governance
Rohwer, Pfadenhauer [55] Integrate-HTA Countries.	T/G	Journal Article										Political and geographical factors, international policies, intervention design and delivery, implementation, Logic models, interactions, relevant evidence, Stakeholder input
Brereton, Wahlster [46] Integrate-HTA Countries.	T/G	Demonstration of Guidance										Political and geographical factors, implementation feasibility, Stakeholder input, interactions, transparent evidence
Lysdahl, Mozygemba [49]	T/G	Guidance										Political and geographical factors, early stakeholder input, interactions, available and informative evidence
Lysdahl, Mozygemba [48] Integrate-HTA Countries.	T/G	Journal Article										Implementation, early stakeholder input, interactions, available and informative evidence.
Lysdahl, Oortwijn [50] Integrate-HTA Countries.	T/G	Journal Article										Principlism, early stakeholder input, interactions, data availability
Lysdahl and Hofmann [47] Integrate-HTA Countries.	T/G	Journal Article										Interactions, adaptability, early stakeholder input
Rehfuess and Gerhardus [24] Integrate-HTA Countries.	T/G	Journal Article										Environmental and geographical factors, interactions, stakeholder advisory panels, data availability, quality and interpretation, implementation.
Rehfuess, Booth [53] Integrate-HTA Countries.	T/G	Journal Article (Synthesis method)										Logic models, early stakeholder input, interactions, timely integration of data
Mathes, Antoine [13] A global perspective	T/G	Journal Article (A Sys. Rev. of the methodological guidance)										Intervention sustainability, relevant evidence, interaction and flexibility. HTA-core domains were not explicitly referenced but the emphasis on context suggests a consideration of these factors.
Potter, Avard [58] Canada	T/G	Guidance (a discussion paper)										Political factors, literature synthesis, expert advice, public engagement, stakeholder values, contextual and transparent evidence, technology-society relationship.
Besley, Henderson [73] England and Wales	T/G	Consulting Report (incl. expert opinions)										Real-world evidence and surrogate endpoints, uncertainty, expert opinion
Elvidge, Summerfield [74] Multi-country perspective (across Europe and North America)	T/G	Journal Article (Policy and methodological guide)										Transparent data, stakeholder input, adaptability, real-world evidence

Green represents “yes”, red represents “no”, and orange represents “possible”.

T/G: Theory or Guidance including those rooted in Integrate-HTA Project

Sys. Rev.: Systematic Review.

**Table 3:** Used evaluation criteria and domains in health technology assessment of complex interventions (adapted from Practical or Empirical Research).

Source & Country	Approach	HTA Product or Dissemination Method	Evaluation Criteria, Domains or Evidence Types Based on the HTA Core Model									Other consideration
			Health problem & current use of technology	Description & Technical Characteristics of technology	Safety	Clinical Effectiveness	Cost & Economic Evaluation	Ethical	Organisational	Patient & Social	Legal	
Polus, Mathes [60] Global perspective	P/R	Journal Article (Practice-based empirical research)										Intervention integrity, sustainability, heterogeneity, stakeholder input, interactions, data availability
Bond and Weeks [59] Canada	P/R	Journal Article (rooted in Integrate-HTA)										Policy context, provider preference, lack of education, stakeholder input, interactions, relevant evidence
Medical Services Advisory Committee (MSAC) [63] Australia	P/R	Full HTA										Cultural safety, stakeholder input, agility, geographical factors, available and relevant evidence.
Medical Services Advisory Committee (MSAC) [64] Australia	P/R	Full HTA										Cultural safety, stakeholder input, consumer feedback, available and relevant evidence.
HIQA [62] Ireland	P/R	Full HTA										Cultural safety, public consultation, uncertainty, geographical factors, stakeholder input, interactive HTA, evidence availability.
Bee, Bower [65] The UK	P/R	Sys. Rev.										Difficulty in measuring outcomes, political factors, interactions, data availability, stakeholder input.
Tappenden, Campbell [66] The UK	P/R	Sys. Rev.										Outcome uncertainty, interactions, data availability, quality and interpretation
Banerjee and Argáez [68] Canada	P/R	Limited Sys. Rev. (Rapid Rev.)										Implementation factors, stakeholder input and preferences, geographical heterogeneity, available evidence
Chen, Yuan [61] China	P/R	Journal Article										
Clark and Sousa [69] Canada	P/R	Realist Review; Perspectives and Experiences Review										Stakeholder input, flexibility, adaptability, transparency in information flow and data, post-implementation evaluation, interactions and evidence gaps
Hui, Dolcine [70] Canada	P/R	Environmental Scan										Staff & patient preparedness, political factors, intervention adoption and implementation, stakeholder input, interactions, evidence quality, availability and relevance.
Hogervorst, Vreman [7] Multi-country study, conducted across European HTA organisations	P/R	Journal Article (Health Policy Analysis)										Quality and quantity of data, outcome interpretation, political pressure, early stakeholder dialogue, advocacy groups
Hogervorst, Pontén [75] Multiple countries across Europe	P/R	Journal Article (Empirical)										Communication, stakeholder collaboration, real-world data, quality data
Lahue, Baginska [67] International Perspectives	P/R	Journal Article; Sys. Rev.										Use of adjunctive technologies and innovative solutions, evidence quality and sufficiency
Carey [76] Ireland	P/R	Thesis (Empirical research incl. a sys. Rev.)										Difficulty in assessing intervention’s long-term effects, uncertainty about additional treatment needs, discrepancies in expert opinions, using systematic literature reviews and expert elicitation
Nurchis, Riccardi [77] Representing HTA organisations in UK, Sweden, Belgium and Canada	P/R	Journal Article (Scoping Review)										Intervention sustainability

Green represents “yes”, red represents “no”, and orange represents “possible”.

T/G: Theory or Guidance including those rooted in Integrate-HTA Project.

P/R: Practice and Empirical Research

Sys. Rev.: Systematic Review

According to this table, for complex interventions, all HTA core domains are considered in the Integrate-HTA methodological guide (i.e., collection of all sub-guidances presented together), MSAC’s full HTA of lung cancer and cervical screening programs in Australia, HIQA’s full HTA of public access defibrillation in Ireland, Bond and Weeks’s experience of the Integrate-HTA and Hui et al.’s Environmental Scanning of virtual care in primary care in Canada. However, other products concerning complex interventions only addressed a subset of the HTA core domains as classified by Busse, Orvain [40] and the EUnetHTA Joint Action 2 Work Package 8 [78].

A summary of these findings is given in Table 4. Except for the technology’s description, its current/intended use, economic impact and organisational aspects, the other assessment criteria or domains (including safety and, ethical, social and legal aspects), were more commonly addressed in the guidance documents than in actual practice (shown in percentage). Clinical evidence was always considered in theory and practice.

**Table 4:** Frequency distribution of assessment criteria or domains in HTA of Complex Interventions in theory and practice.

Assessment Criteria, Domains or Evidence Types	Guidance (n = 19)	Practice (n = 16)	Total
Health problem targeted by technology and its current/intended use	14 (74%)	12 (75%)	26
Technology’s description and technical characteristics	13 (68%)	12 (75%)	25
Safety	15 (79%)	12 (75%)	27
Clinical effectiveness	19 (100%)	16 (100%)	35
Economic impact incl. costs and economic evaluation	18 (95%)	16 (100%)	34
Ethics	17 (89%)	11 (69%)	28
Organisational aspects	13 (68%)	15 (94%)	28
Patient and Social aspects	18 (95%)	12 (75%)	30
Legal aspects	14 (74%)	7 (44%)	21

• This table exclusively presents the ‘yes’ responses.

• Ethics, organisational, patient, social and legal aspects are areas with significant discrepancy between theoretical expectations and practical implementation.

We also identified the following domains or factors that were suggested for inclusion or reported in HTAs of complex interventions.

- *Politics*: This domain focuses on the impact of political factors (such as government priorities and lobbying) on the assessment and decision-making processes related to health technologies. Tables 2 and 3 show 11 records (eight guidance documents and three actual studies) highlighting *political* factors in the HTA of complex interventions.
- *Implementation* and *feasibility*: This domain refers to the practical execution of an intervention in real-world settings or the action that must follow any preliminary thinking for an intervention to occur. Tables 2 and 3 show nine records (six guidance documents and three actual studies) highlighting *implementation/feasibility* in HTA of complex interventions.
- *Sustainability*: This domain focuses on the intervention’s ability to continue having its intended impact over time. It includes considerations of financial, environmental and operational sustainability to ensure the intervention’s long-term effectiveness and functioning. Tables 2 and 3 show four records (two guidance documents and two actual studies) highlighting s*ustainability* in HTA of complex interventions.
- *Outcome logic and mechanisms of change/impact*: This domain explores how an intervention works and how it will improve health outcomes. Tables 2 and 3 show six records (three guidance documents and three actual studies) highlighting these factors in HTAs of complex interventions.
- *Data quality and availability:* This domain emphasises the importance of having relevant data including contextual and real-world evidence to inform HTA of complex interventions. High-quality, available data ensure that assessments are well-rounded and consider all relevant factors, not just clinical or technical data, thereby enhancing the accuracy and relevance of the evaluation. Tables 2 and 3 show 32 records (18 guidance documents and 14 actual studies) highlighting data availability, quality and interpretation.
- *Geographical variability:* This domain shows how regional differences in infrastructure and needs may affect intervention effectiveness and implementation. If HTA processes focus solely on aggregate data without accounting for regional variations, interventions might not be well-suited for specific areas. Tables 2 and 3 show that geographical context is a focal point in 10 papers that allude to the Integrate-HTA project and in three actual studies.
- *Interaction and Stakeholder Engagement:* The complex interplay of contextual factors, implementation processes and diverse stakeholder characteristics emerged as a pivotal focus in many documents. For instance, interactions may encompass how cultural norms affect healthcare technologies’ impact. These dynamic, interdependent influences are crucial for effective HTA of complex interventions and informed decision-making. Notable among these interactions is the central role of stakeholders, including patients, their active participation, diverse perspectives and vested interests, in shaping CI HTA processes and decisions. Tables 2 and 3 indicate 30 records (18 methodological papers and 12 actual studies), underlining the need to understand these intricate, interdependent influences when conducting HTAs for complex interventions.
- *Dimension of Time*: Five records also expressed concerns about the challenges in assessing complex interventions’ long-term effects and uncertainties surrounding their true value, potential outcome changes, or additional treatment needs over time [62,76].

### Comparative perspectives on HTA of complex interventions

There is a divergence between theoretical concepts and frameworks proposed to guide HTA of complex interventions and their actual application or practical implementation. Different countries prioritise different aspects of HTA when evaluating complex interventions in real-world settings:

#### Theory-driven assessment.

This perspective, largely based on established theories and guidelines, prescribes guidance for conducting HTA of complex interventions through a multi-stage process involving multidisciplinary teams and encompassing many interconnected components within the intervention and system environment. It is used in Integrate-HTA and is most common in European countries, particularly those that use structured methodologies and established guidelines to evaluate complex technologies [5]. These countries have established agencies responsible for conducting HTAs, and these agencies often produce guidance documents to ensure consistency and transparency in the assessment process.

The Integrate-HTA model offers a broader perspective and different assessment process for complex interventions than the HTA core domains and aims to adapt and develop HTA concepts and methods to enable patient-centred, integrated assessments of the effectiveness and economic, social, cultural, epidemiological, geographical, political and ethical issues of complex interventions while accounting for patient preferences and patient-specific “moderators” of treatment, setting and implementation (i.e., provider, organisation and structure, funding and policy) [72]. The Integrate-HTA model arguably enables a coordinated assessment of all these domains and their interdependence and incorporates stakeholder perspectives to produce results. The model proposes that integration should begin early in the process, stakeholders should be involved and traditional HTA methodologies should be adapted to allow for integrated assessments [79]. The Integrate-HTA process is structured into five stages [24,79]:

1. *Defining the technology under assessmen*t and *clarifying the HTA’s objective(s)* with stakeholder input;
2. *Developing an initial logic model* to provide a structured overview of the intervention and the system in which it is embedded, helping to conceptualise the relationship between intervention, context and desired outcomes;
3. *Systematically compiling and assessing evidence* on effectiveness and economic, ethical, socio-cultural, legal and other aspects, while considering diversity of participants, implementation contexts, and interactions*;*
4. *Structuring assessment results by populating an extended logic model* with data generated in the previous step, visualising relationships between all intervention features, contexts and outcomes of interest, and identifying gaps in the evidence. This step helps contextualise and communicate the integrated assessment findings to inform decision-making.
5. *Drawing conclusions and formulating recommendations* to facilitate decision-making. This step, not an integral part of the HTA, involves deliberative decision-making using quantitative methods like Multi-Criteria Decision Analysis or qualitative aids.

#### Real-world assessment.

This perspective, purely empirical, demonstrates how HTA of complex interventions is implemented and applied in different contexts. Certain domains and criteria proposed in theoretical guidance may not be fully employed in practice, as reflected in the actual HTA reports, and this varies by country. In practice, HTAs of complex interventions tend to strongly emphasise clinical, economic and organisational data, particularly parameters like cost and clinical effectiveness. There is a gradual, albeit moderate, shift toward considering aspects related to technology description, current use and safety, as well as patient and social factors. However, ethical and legal dimensions often receive comparatively less scrutiny within these assessments.

This scoping review found that the assessment of complex interventions exhibited slight variations across the selected countries, with many using similar overarching domains and evaluation criteria but placing varying degrees of emphasis on them or prioritising certain aspects over others during the assessment process.

##### Canada:

This scoping review identified four Canadian Agency for Drugs and Technologies in Health (CADTH) documents encompassing HTAs of a complex intervention in Canada: a limited systematic review [68], a realist review [69], an environmental scan [70], and an empirical study on the application of Integrate-HTA guidance in Canada [4].

CADTH uses a concurrent HTA approach to evaluate complex health interventions, where multiple assessments are carried out simultaneously to assess the various aspects of the intervention. Using the CADTH Health Technology Expert Review Panel Deliberative Framework, a health economist, an ethicist, a public member, three clinical experts, and up to five additional experts for specific purposes will evaluate interventions and provide HTA recommendations. Ten domains of diverse value streams define the information the panel needs from an HTA to guide their deliberations (Table 5). This process requires domain-specific research questions, specialised research teams, and regular meetings to incorporate feedback [4].

**Table 5:** CADTH health technology expert review panel deliberative framework.

Domain	Examples of aspects considered
Background	Audience; issue and policy question
Need	Health condition, affected population, available alternatives
Benefits and harms	Efficacy, safety, clinical effectiveness, impact on patient-centred outcomes and clinical management; non-health benefits (e.g., patient autonomy, dignity)
Patient preferences	Experiences with condition and technology; acceptability of technology
Economic impact	Cost-effectiveness, budget impact, infrastructure support costs
Implementation	Healthcare processes, workforce, integration of technology into existing workflow, training/competency requirements, repair and maintenance
Legal impacts	Legal or regulatory issues
Ethical issues	Consistency with Canadian ethical values
Environmental impact	Resource use, recyclability, waste disposal
Other	Issues not raised above

##### Ireland:

 This scoping review found one full HTA of an Irish public access defibrillation program [62] and a PhD thesis-based HTA focusing on specific cancer treatments [76], both representing complex interventions. Ireland appears to follow more of a sequential approach to HTA for complex interventions rather than a fully concurrent approach. The process typically involves separate, sequential phases for assessing different aspects of a health technology or intervention. In the evaluation of the public access defibrillation program, Ireland’s Health Information and Quality Authority (HIQA) highlighted a series of key components and domains of value as follows:

- Prioritisation and topic selection
- Developing the HTA’s Terms of Reference and reaching an agreement with the Department of Health. This included setting standards for health and social services, focusing on evidence and best international practices;
- Creating an Expert Advisory Group and evaluation team of internal staff to assist with ethical and legal analyses and out-of-hospital cardiac arrest incidence data for the economic model;
- Conducting an evidence-based systematic review to understand the program’s effectiveness and safety;
- Reviewing Irish epidemiological data and relevant international literature on out-of-hospital cardiac arrest;
- Analysing data on Ireland’s public automatic external defibrillators and examining the configuration of emergency medical services;
- Determining the cost-effectiveness of different public access defibrillation program configurations;
- Reviewing the program’s broader effects on patients, communities, and the healthcare system, including its ethical, legal, and social implications;
- Drafting the report and inviting public comment before finalising it;
- Providing advice on the optimal configuration of the program, i.e., design, implementation, and operation.

In Irish HTA of complex interventions, expert opinion is enlisted to bridge gaps where data or evidence may be lacking.

##### Australia:

This scoping review found two full-scale HTAs of complex interventions in Australia (lung cancer and cervical screening programs) conducted by the Medical Services Advisory Committee (MSAC) and following Commonwealth HTA processes [63,64]. The Australian approach to HTA for complex interventions appears to be more of an integrated concurrent model, though there are still some sequential elements. For complex interventions, the MSAC process involves concurrent evaluation of different evidentiary domains by a team of evaluators, working with - or advised by – relevant clinicians and public health experts, separate sub-committees or working groups tasked with identifying implementation considerations, and which then feed into an overall integrated appraisal by MSAC. The HTA of a lung cancer screening program, e.g., included the following components and domains of value:

- Developing Terms of Reference for the independent assessment of the intervention’s comparative safety, effectiveness, cost-effectiveness, and total cost based on the best available evidence;
- Reviewing national and international evidence to understand the program’s benefits, harms, eligible population, cost-effectiveness, best-practice clinical pathways, and key elements of the targeted program; the review also included prerequisites essential for implementing any funding advice, ensuring adherence to regulatory compliance standards, thereby establishing a robust framework for financial decisions.
- Estimating the number of lung cancer cases in Australia attributed to various risk factors to inform program eligibility criteria;
- Examining the sociodemographic characteristics of Australians who died from lung cancer to identify priority groups for targeted communication in the program, with the aim of addressing clinical benefits (effectiveness) or equity of access (ethics);
- Conducting an economic evaluation to understand the economic costs, benefits, and outcomes of the program; the safety and effectiveness of the screening tests were assessed through randomised controlled trials (RCTs).
- Designing the screening program and clinical assessment pathway to ensure equitable access for all Australians; Involving stakeholders, including patients, clinicians, industry representatives and health system decision-makers, to get their opinions on the proposed screening program. Consultations entailed deliberating on potential changes to the screening pathway.
- Preparing a comprehensive report summarising the HTA findings, recommending the optimal design, implementation and operation of the screening program, and disseminating it to relevant stakeholders and decision-makers;
- Implementing and monitoring the recommended screening program to ensure its success.

#### How does Australia’s HTA of Complex Interventions compare with Canada’s, Ireland’s and Integrate-HTA?

Table 6 provides an overview of how Australia, Canada and Ireland practise HTA for complex interventions, summarising differences in their processes and the degree of integration and concurrency in their evaluations.

**Table 6:** Comparison of complex interventions HTA in Australia, Canada, and Ireland.

Country	Approach to HTA of complex interventions	Explanation of Process
Australia	Integrated, concurrent	Australia uses an integrated concurrent model, allowing simultaneous evaluation of different evidentiary domains, e.g., clinical, economic and ethical factors, by a team of evaluators advised by relevant professionals and public health experts. Separate sub-committees or working groups identify implementation considerations, feeding into an overall integrated appraisal by the Medical Services Advisory Committee.
Canada	Concurrent, transitioning to integrative	Canada is transitioning towards a more integrated HTA model, although the process is predominantly concurrent. The CADTH Health Technology Expert Review Panel uses a deliberative framework involving multiple experts to evaluate interventions and provide recommendations.
Ireland	Sequential with some concurrent elements	Ireland traditionally follows a more sequential HTA approach, where assessments are completed in phases, although some overlap exists. Often, clinical and economic evaluations are completed before moving on to other aspects, such as ethical, social, and organisational impacts. Expert opinion is enlisted to bridge gaps where data or evidence may be lacking.

Australia’s approach to evaluating complex interventions in healthcare is not purely concurrent - integration happens across MSAC’s process; it falls between a sequential and fully concurrent model, leaning towards an integrated approach. An evaluation group conducts an initial PICO (Population, Intervention, Comparator, Outcome) analysis, which is then considered by the PASC (Protocol Advisory Sub-Committee). PASC evaluates the place of the technology in clinical practice and implementation considerations. Following this, an HTA is conducted by evaluators such as the Australian Health Technology Assessment (AHTA) team. Simultaneously, policy and implementation issues are determined by the government. The HTA findings and policy considerations are subsequently reviewed by the Evaluation Sub-Committee (ESC) and then submitted to the Medical Services Advisory Committee (MSAC) for final deliberation (i.e., the ESC appraises the safety, clinical effectiveness and economic considerations, and equity, ethical, organisational and access issues, as needed, on the basis of the combined independent evaluation in the HTA report. This process ensures that clinical effectiveness, economic considerations and equity issues are comprehensively evaluated in a coordinated manner. Policy and programmatic considerations are then summarised for MSAC appraisal by the relevant policy area in government. MSAC synthesises these inputs into an integrated appraisal and funding recommendation, capturing the interplay between different factors tailored to the complexities of multifaceted health interventions). While there is still a sequential element in the appraisal, in that the PASC review occurs before the ESC review, a concurrent process occurs during the evaluation stage. This approach leverages parallel assessments and integrative synthesis for a holistic assessment of multifaceted interventions in Australia’s healthcare system.

Ireland traditionally follows a more sequential HTA approach, although there are instances where certain assessment phases overlap or run concurrently to some extent. Assessments like clinical and economic evaluations are completed before moving on to other aspects such as ethical, social and organisational impacts. For complex interventions, HIQA guidelines show that the different assessment strands may need to be more closely integrated and conducted in parallel to capture the relationship between components. Nevertheless, a fully integrated, concurrent model does not yet seem to be standard practice across all HTAs in Ireland.

Canada’s HTA process is predominantly concurrent, assessing various aspects of an intervention or treatment simultaneously. However, the process is transitioning to a more integrated HTA model that concurrently examines all facets, albeit with anticipated challenges. This integrative approach by CADTH aims to synthesise various assessments and considerations into a holistic evaluation.

In all three countries, Expert or Scientific Advisory Groups, comprising key stakeholders and HTA methodological experts, review evidence, provide input and follow established guidelines to produce independent assessment reports. Expert opinion is sought for clarification where data are missing. The three countries provide independent, evidence-based recommendations concerning the target population. They feature stakeholder engagement and have well-established regulatory, funding and reporting structures to guide the assessment and approval of health innovations, but each country weighs factors differently. Stakeholders are most engaged during the appraisal stage, especially in evidence evaluation and recommendation generation, though their composition, type, level of involvement and prioritisation in engagement activities vary by stage.

Approaches like Integrate-HTA seek and advocate for stakeholder input from the beginning of the HTA process, i.e., the scoping and research question design phase. This early involvement aims to strengthen the comprehensiveness of the assessment. In practice, however, CI HTA processes often incorporate stakeholder input to varying degrees while prioritising clinical, economic and organisational evidence over other domains. The challenge is to properly balance comprehensive stakeholder engagement with the need for rigorous evidence-based assessment across all relevant domains. The real-world practices also exhibit context-specific variations in criteria rather than strict adherence to multi-component theoretical frameworks across all settings.

This scoping review found that integrative approaches to HTA give equal weight to core assessment domains for complex interventions, in line with the theoretical HTA guidance; however, concurrent and sequential models, even while they may assess the same domains, tend to give unequal weight, emphasising some domains over others. For example, clinical and economic factors have traditionally taken precedence in concurrent and sequential assessments.

Nevertheless, there is growing recognition of the importance of also evaluating ethical, social, legal, implementation and other contextual factors and interactions, particularly for complex interventions [20,29]. Australia’s integrated concurrent approach for complex interventions and Canada’s move towards integrative HTA aim to holistically capture the interplay between clinical, economic, ethical, socio-cultural, legal and feasibility considerations when evaluating complex interventions. While clinical and economic evidence remains crucial, a clear trend is emerging towards prioritising and integrating a more comprehensive range of assessment domains for complex interventions across HTA bodies in Australia and Canada.

## Discussion

This scoping review examined 35 publications to determine the domains of value and criteria used and recommended for use in HTAs of complex interventions, as well as exploring the approach to the HTA of complex interventions.

### Evidence type: domains and evaluation criteria

This scoping review found a range of domains and evaluation criteria in the HTA of complex interventions. The domains and criteria fell into two broad categories: those rooted in methodological guides and theoretical literature and those used in practice. While there was agreement in the methodological literature on what evidence and evaluation criteria should be used, in practice each domain was given uneven emphasis or prioritisation.

The unequal weighting given to HTA domains and evaluation criteria may signify a deliberate prioritisation of specific domains/criteria as more critical or influential in shaping the overall funding recommendation. Actual HTA studies included in this review, largely concurrent, emphasised economic evaluation (i.e., cost-effectiveness), clinical effectiveness and organisational impacts as primary domains when assessing a complex intervention, with a gradually increasing emphasis over time, on technology’s intended use, description, safety, social aspects and patient preferences, and less information on ethical and legal considerations. This prioritisation might stem from the perceived importance of such data in specific healthcare contexts or the need to inform a funding, as opposed to an implementation, decision. There may also be a tendency to prioritise these domains due to established methods or mandates [80], data availability, unique contexts and needs, and the more tangible nature of economic and efficacy measures compared to assessing ethical or societal impact. For complex interventions in particular, social, legal and ethical factors can be more challenging to systematically evaluate given their context-dependent, qualitative and value-laden nature [e.g.,^58^,^76^]. Such prioritisation decisions, however, can result in unbalanced evaluation outcomes, potentially overlooking critical aspects of interventions that might affect successful implementation. This highlights the complexity and challenge of achieving a comprehensive assessment that adequately considers the multifaceted nature of complex interventions.

Methodological guidance, such as Integrate-HTA, provided equal weight to the HTA core domains of safety, effectiveness and cost-effectiveness [40], context-specific aspects (including socio-cultural, ethical, legal, and political), implementation issues, patient preferences and stakeholder engagement [71,81]. Likewise, full HTAs of complex interventions in Australia and Ireland, as well as CADTH’s experience in adapting Integrate-HTA to Canada, emphasised the HTA core domains. However, other products, such as systematic and realist reviews, address only a subset of these factors, with cost and clinical effectiveness aspects prevailing, and other factors given varying degrees of emphasis.

The methodological guidance, full HTAs and CADTH’s environmental scan emphasised context, viewing interventions as “events in systems” [82] which interact with features of contexts to produce effects [83]. This suggests that complex interventions, in particular, cannot be evaluated independent of their context. Context is highly influential in shaping the mechanism and outcomes of complex interventions [82]; however, comprehensive consideration of all relevant contextual aspects, e.g., ethics or politics, remains infrequent, especially in actual HTAs. It may be that as HTA concentrates on new technologies, primarily before they have been embedded in the health system, all of these contextual factors cannot be anticipated and assessed.

Logic models were introduced as helpful tools for identifying contextual factors and potential causal pathways in the HTA of complex interventions [9,53]. However, these models have been criticised for being too theoretical, resource-intensive and time-consuming to develop; for not adequately representing real-world complexities such as the diversity of components, non-linearity, relationships and pathways; and for perpetuating the status quo because they frequently reflect the perspectives and interests of those who create them [e.g., 84]. Other valid critics, however, argue that it is unfair to criticise logic models for failing to capture the dynamics and interrelationships of contextual variables influencing outcomes, as this goes beyond their intended purpose. They argue that logic models serve to logically summarise the alignment of conditions, strategies, and measurements in a program, highlighting its focus, which may only depict the narrow boundary of elements targeted by that program [85]. Incorporating system thinking concepts and multiple stakeholder perspectives into logic models from the outset may allow for more meaningful and valuable assessments of complex intervention dynamics and interrelationships [85,86].

Incorporating implementation aspects, ethics, patient preferences and stakeholder engagement into the HTA of complex interventions were also highlighted as crucial for a comprehensive assessment. Incorporating implementation factors (such as resource availability, intervention feasibility and the capacity of the healthcare system to implement the intervention effectively) helps to identify potential obstacles to implementation and informs decision-making about the intervention’s use in practice. Likewise, incorporating multiple perspectives, opinions and values of stakeholders, including patients and providers, can significantly improve the intervention’s relevance, acceptability, uptake and effectiveness [83,87]. As HTA moves increasingly into health technology management, these elements will become more important [88].

Implementation aspects, ethics, patient preferences and stakeholder engagement were strongly emphasised in methodological guides; however, the extent to which these domains were included in the actual HTA of complex interventions varied. Limited focus on these factors can lead to interventions that are not well-suited to local contexts, making them less effective or less likely to be adopted and sustained. It can also result in inaccurate conclusions regarding the benefits and risks of the intervention.

This review found little emphasis on post-implementation evaluation or adaptability to change, particularly regarding funding decisions. Post-implementation evaluation can provide valuable insights into the implementation process and the intervention’s impact on intended or target populations. It is critical for demonstrating impact and understanding the factors that determine intervention success or failure. In addition, adaptability to change allows the intervention to evolve, ensuring that the intervention remains relevant and effective [89]. Failing to consider these aspects may result in an incomplete understanding of complex interventions and whether their initial promise has been realised.

Our review found that most HTA reports on complex interventions (>94%) focused on cost and economic evaluation, clinical effectiveness, and organisational aspects, with progressively increasing, though moderate (around 75%) emphasis, on technology’s intended/current use, description and technical characteristics, safety, social aspects, and patient preferences, but less emphasis on ethical and legal considerations.

In a survey of INAHTA members a decade ago [43], the four most common HTA products (full HTA, mini-HTA, rapid review and policy brief) typically included a description of the technology, an overview of its current use, and information on its safety and effectiveness, followed by an economic evaluation. The organisational domain was largely addressed in full HTA reports and sometimes in mini-HTA reports. Full HTAs were more likely to include information on the ethical, social and legal core domains than other HTA products. The ethical, social and legal domains had the lowest inclusion rates across all HTA product types.

Compared to the results of this survey, for the most part, complex interventions are treated the same way as other technologies in HTA, albeit with the inclusion of some other domains used to varying degrees. Political considerations, implementation and feasibility, sustainability, outcome logic and process change/impact mechanisms, data quality and availability (including expert opinion), geographical variability, as well as stakeholder interaction and engagement emerge as other critical domains for evaluating complex interventions. While sometimes overlooked in the conventional HTA framework, these domains have been acknowledged as instrumental in capturing the nuanced interplay between complex interventions and their operational environments [7,90,91]. Incorporating these domains into the evaluation process signifies the evolving landscape of HTA, reflecting a recognition of the contextual complexities within which interventions are introduced and managed. The rationale for their inclusion stems from the recognition that complex interventions, by their very nature, interact with intricate contextual dynamics that extend beyond traditional HTA parameters. These variations may stem from differences in study design, time period and the specific health technologies included in the analysis.

### Approaches to HTA of complex interventions: integrative, sequential and concurrent perspectives

This scoping review identified three distinct approaches to the HTA of complex interventions: the integrative approach, which is emphasised in methodological guides and theoretical literature, and the concurrent and sequential approaches, which are emphasised in practical HTAs, such as the full-scale HTA publications and environmental scans.

*i) The integrative HTA review approach* recognises that complex interventions have multiple interrelated components, considers the interconnections and interdependencies between them, and seeks to evaluate the complex intervention in a holistic and interdisciplinary manner.

The Integrate-HTA model is a one-of-a-kind exemplar of an integrative approach, offering a framework with a five-step process for the HTA of complex interventions. However, while the guide is comprehensive and theoretically sound, there has been some debate about its practical application. CADTH’s experience, for example, highlights the challenges of integrating multiple methods and disciplines cohesively and operating in resource-constrained environments [4]. According to the authors, it is practically “impossible to assess different, interacting aspects independently and then attempt to complete the integration afterward” [4]. The framework is complex and time-consuming to apply, requiring significant resources and expertise [4]. This might represent a theory-practice gap, which may occur when HTA researchers and methodological experts struggle to apply their academic knowledge to the complexities of real-world decision-making practice, such as when integrating multiple data sources and considering different stakeholders’ perspectives. Bridging this theory-practice gap requires collaboration between researchers, practitioners and decision-makers to ensure that HTA guides and results are relevant and valuable in the real world.

It is worth noting that there is often an inherent tension between making a framework more theoretically sound and making sure it can actually be used in practice. Balancing theoretical rigour with pragmatic real-world applicability may pose an ongoing challenge for integrative HTA approaches like Integrate-HTA when evaluating highly complex interventions.

*ii) The concurrent HTA review approach*, predominant in Australia and Canada, seeks to maximise the strengths of different methods and provide a comprehensive evaluation of the intervention. It tends to evaluate individual components or aspects of the intervention at the same time but sometimes in isolation [25,80,90,92,93]. As a result, decision-makers must combine, synthesise and interpret the various pieces of evidence and information to reach a final judgement, potentially highlighting the need for improved integration of the various information domains and stakeholder perspectives within the HTA process [25]. It is possible that the pragmatic concurrent gathering of evidence on complex interventions stems from the necessity of producing timely findings.

However, segregated evaluations can make it difficult to capture the interplay and emergent effects arising from complex interventions. An integrative synthesis step is likely required to holistically interpret the collective HTA findings within the broader context and system environment of the complex intervention. While the concurrent stream allows simultaneous assessments by specialised teams or committees to expedite the overall HTA process, it may also lead to challenges in ensuring cohesive integration and synthesis of the diverse pieces of evidence.

It is essential, however, to differentiate between the role of the assessor/evaluator and the decision-maker. The HTA agency may lack the mandate to mobilise the value judgements required for the integration of information. It may be that decision makers need more tools or support in doing the integration, since they will need to judge, for instance, the need to prioritise ethics over clinical effectiveness in a particular case. It may lie beyond the purview or responsibility of the HTA evaluator to perform this task of weighing and synthesising evidence across domains based on context-specific value judgements.

Concurrent approaches provide valuable information and have the potential to speed up the HTA process, but they are often criticised for *limited stakeholder engagement*, in which stakeholder views and perspectives may not be fully considered, particularly in terms of scale and timing of engagement. Unlike the Integrate-HTA, engagement often occurs at a later stage of the evaluation process, after the initial analysis and assessment have been completed, limiting opportunities for stakeholders to provide input and feedback on the intervention and the evaluation process, influencing the evaluation results [94].

*iii) The sequential HTA review approach*, predominant in Ireland, follows a step-by-step evaluation process where assessments are conducted sequentially, one aspect at a time. Unlike concurrent approaches that evaluate multiple components simultaneously, the sequential model focuses on thoroughly examining each dimension before progressing to the next. This systematic sequential approach aims to provide a comprehensive evaluation by dedicating full attention and time to each assessment component.

Proponents argue that this method leads to more rigorous analyses compared to concurrent evaluations, which may overlook key details when spreading resources across multiple concurrent streams. While prioritising thoroughness, the sequential approach may prolong the evaluation timeline as each phase must be completed before moving on. The extended durations of sequential reviews may limit the capacity to holistically evaluate emergent systemic impacts stemming from the inherent complexity of integrated/multifaceted interventions. Critics also suggest that the sequential approach’s compartmentalised reviews cannot fully capture the interrelated, dynamic effects arising from the complexity of integrated interventions and could delay the delivery of assessment findings needed for timely decision-making.

A comparison of the three approaches reveals some divergence between the theory and practice of HTA of complex intervention. Theoretical underpinnings advocate for a holistic view of considering a broad spectrum of factors and their relationships. However, pragmatic implementation often tends to focus on specific domains or aspects of complex interventions, potentially overlooking other critical components. One possible explanation is the difficulty in effectively translating theoretical concepts into actionable real-world applications. Besides, the gap between theory and practice is compounded by the different priorities placed by various countries on different aspects of CI HTA, adding a deliberative layer.

Achieving a balance between comprehensive evaluative scrutinies and delivering integrated actionable insights within practical timeframes remains an ongoing methodological challenge when assessing complex interventions. Reconciling the need to thoroughly examine each part with system-level contextual realities and the bigger picture of how everything fits together can be difficult and poses inherent tensions. An agile framework balancing theoretical robustness with pragmatic feasibility may be required to enable consistent, high-quality complex intervention evaluations across diverse settings.

Adopting a complex systems thinking approach and actively incorporating diverse stakeholder perspectives into logic models from the outset may allow for more comprehensive and valuable assessments of complex interventions. By considering implementation aspects, contextual factors, plans for post-implementation evaluation and the need for adaptability early in the process, assessors can better capture the dynamic interrelationships and emergent properties that characterise complex interventions. This proactive integration of systems-level factors and multidisciplinary viewpoints into the conceptual models guiding the assessment allows for a deeper understanding of the non-linear dynamics, feedback loops and contextual influences that shape the outcomes and impact of complex interventions within complex adaptive healthcare systems [95]. Such an approach has the potential to yield more meaningful and actionable insights, ultimately informing more effective strategies for the successful implementation and sustainable adoption of complex health interventions. Whether this can be done in a timely manner is, however, uncertain.

Complexity thinking provides a promising framework for HTA of complex interventions through its holistic approach to understanding interventions and their surrounding systems. This approach acknowledges the dynamic and interconnected realities of the intervention context, moving beyond traditional linear cause-effect analysis to address the multifaceted interactions within systems including the multifactorial influences on outcomes, like stakeholder interactions, organisational structures and adaptation processes [26,27]. By embracing this systems approach, HTA can more accurately capture and evaluate the intricate realities of complex health interventions. For example, when assessing an integrated care pathway, complexity thinking encourages assessors to map and explore the interplay of elements—e.g., clinical protocols, staff training, IT systems, and patient engagement—ensuring they’re assessed as a cohesive and unified whole rather than as isolated components. This facilitates identifying key implementation factors, like how staff adaptations to change, e.g., new workflows, impact patient outcomes or how organisational structures can support or impede the intervention’s success. Stakeholder analysis, guided by complexity thinking, can also help assessors understand how diverse settings/contexts, such as urban versus remote health, might benefit from distinct implementation strategies for the same intervention. Such an understanding supports assessors in developing tailored evidence-collection methods, such as combining quantitative outcome measures with qualitative process evaluations, to capture immediate and evolving impacts. Complexity thinking enables HTA evaluations to produce more context-sensitive and actionable insights through these practical applications, helping health policy and decision makers anticipate challenges across varying contexts and make informed decisions.

Yet, operationalising the complex systems thinking approach in HTA of complex interventions, particularly incorporating stakeholder engagement, logic models and systems thinking in evaluations, remains a significant challenge. These aspects are widely advocated in theory; however, their practical implementation requires clear guidance.

Approaches such as realist evaluation and established HTA processes (e.g., those followed by MSAC in Australia) are argued to offer a practical guide for applying complex systems thinking. Realist synthesis methodology, for example, offers one structured pathway forward, as it systematically examines ‘what works’, ‘for whom’, ‘under what circumstances’ and ‘why’. It incorporates systems thinking by considering the interplay between context, mechanisms and outcomes [96,97]. Likewise, the assessment process used by MSAC in Australia demonstrates how logic models and stakeholder engagement can be effectively incorporated into health technology assessments [see, e.g., 64]. These examples may demonstrate the feasibility of applying complexity thinking in real-world settings. However, it remains unclear how different aspects are systematically integrated into HTA processes for complex interventions. Given this challenge, further research is needed to elaborate on operationalising a systems approach in context.

Our scoping review is possibly the first to synthesise the international literature on CI HTA. However, there were some limitations to the scope and execution of this review. Firstly, we focused only on English literature concerning the HTA of complex interventions, so it is possible that some relevant studies were overlooked or excluded from the review, resulting in a partial representation of the available evidence. Second, some of the included studies only examined single factors, which could limit the breadth of insights and overall understanding of the topic [e.g., 50,68,69,76]. Third, despite our efforts to conduct independent citation screening and data extraction, our narrative synthesis may reflect some degree of subjective appraisal of study findings, potentially introducing bias. Fourth, in our systematic search, we utilised specific terms such as ‘complex’, ‘multi-component’, ‘multi-part’, ‘multifactorial’, and ‘bundled’ interventions, along with variations of ‘program’ or ‘programme’. This approach may not have captured all relevant HTA reports, as these terms are not universally used in the literature, and many relevant studies may be categorised under different terminology. Not all interventions are explicitly labelled as ‘complex interventions’, as this classification is not universally applied. Future research should use a broader range of search terms and synonyms related to complex interventions to ensure more comprehensive literature coverage. Researchers may also explore alternative classification systems and terminologies in HTA reports to improve search inclusivity and accuracy. Developing a more precise and widely accepted definition of complex interventions could also standardise research and enhance the identification and evaluation of relevant studies.

## Conclusion

This systematic scoping review examined the domains, assessment criteria and approaches used and recommended to evaluate complex interventions in HTA globally.

The review identified three distinct approaches to HTA of complex interventions: the integrative approach, emphasised in theoretical guidance, as well as the sequential and concurrent approaches, more prevalent in actual HTA practice in countries like Ireland, Canada and Australia. The sequential and concurrent approaches to HTA for complex interventions may face challenges in capturing interplay and emergent effects, while the integrative approach aims to holistically interpret findings within the broader context. Balancing comprehensive evaluation and timely, actionable insights remains an ongoing methodological challenge, necessitating an adaptable framework that bridges theoretical principles with real-world constraints.

We encourage adopting an integrative approach to the HTA of complex interventions, moving beyond purely concurrent or sequential methodologies. An integrative approach can potentially improve the HTA of complex interventions by simultaneously and systematically integrating and synthesising multiple aspects - including stakeholder perspectives, implementation contexts, system dynamics and emerging feedback loops - within a unified, adaptive framework. While practical challenges such as resource implications and coordination requirements may arise during the integration, the benefits are substantial. An integrative approach is most likely to facilitate real-time responses to emerging issues, promote continuous engagement of stakeholders throughout the evaluation process, and capture interactions inherent in complex systems or interventions. Yet further research is needed to concretise and test this approach in practice.

The scope and specificity of HTA domains varied across reports, with different countries assigning varying weights to various aspects of an HTA. Yet, in real-world HTA practice cost-effectiveness, clinical efficacy and organisational aspects, followed by technology’s description, intended use, safety, as well as patient and social aspects dominated complex intervention evaluations –an improvement over initial approaches over a decade ago. However, ethical and especially legal considerations are still relatively underrepresented in the actual HTA of complex interventions.

The review highlighted the use of diverse additional assessment criteria in HTAs of complex interventions, including political considerations, implementation and feasibility, sustainability, outcome logic and process change/impact mechanisms, data quality and availability (including expert opinion), geographical variability, as well as stakeholder interaction and engagement. The value and weight of this additional information to decision-makers when deliberating on funding recommendations is, however, presently unknown.

## Supporting information

S1 Checklist PRISMA-ScR (Preferred Reporting Items for Systematic reviews and Meta-Analyses extension for Scoping Reviews) Checklist. (DOCX)
